# Novel Molecule Exhibiting Selective Affinity for GABA_A_ Receptor Subtypes

**DOI:** 10.1038/s41598-017-05966-x

**Published:** 2017-07-24

**Authors:** Cecilia M. Borghese, Melissa Herman, Lawrence D. Snell, Keri J. Lawrence, Hyun-Young Lee, Donald S. Backos, Lauren A. Vanderlinden, R. Adron Harris, Marisa Roberto, Paula L. Hoffman, Boris Tabakoff

**Affiliations:** 1 The University of Texas at Austin, Waggoner Center for Alcohol and Addiction Research, Austin, TX 78712 USA; 2 Department of Neuroscience, The Scripps Research Institute, La Jolla, CA 92037 USA; 3 Lohocla Research Corporation, Aurora, CO 80045 USA; 4 Department of Pharmaceutical Sciences, University of Colorado Skaggs School of Pharmacy & Pharmaceutical Sciences, Aurora, CO 80045 USA; 5 Department of Pharmacology, University of Colorado School of Medicine, Aurora, CO 80045 USA; 6 Bowles Alcohol Research Center, Chapel Hill, NC 27514 USA

## Abstract

Aminoquinoline derivatives were evaluated against a panel of receptors/channels/transporters in radioligand binding experiments. One of these derivatives (DCUK-OEt) displayed micromolar affinity for brain γ-aminobutyric acid type A (GABA_A_) receptors. DCUK-OEt was shown to be a positive allosteric modulator (PAM) of GABA currents with α1β2γ2, α1β3γ2, α5β3γ2 and α1β3δ GABA_A_ receptors, while having no significant PAM effect on αβ receptors or α1β1γ2, α1β2γ1, α4β3γ2 or α4β3δ receptors. DCUK-OEt modulation of α1β2γ2 GABA_A_ receptors was not blocked by flumazenil. The subunit requirements for DCUK-OEt actions distinguished DCUK-OEt from other currently known modulators of GABA function (e.g., anesthetics, neurosteroids or ethanol). Simulated docking of DCUK-OEt at the GABA_A_ receptor suggested that its binding site may be at the α + β- subunit interface. In slices of the central amygdala, DCUK-OEt acted primarily on extrasynaptic GABA_A_ receptors containing the α1 subunit and generated increases in extrasynaptic “tonic” current with no significant effect on phasic responses to GABA. DCUK-OEt is a novel chemical structure acting as a PAM at particular GABA_A_ receptors. Given that neurons in the central amygdala responding to DCUK-OEt were recently identified as relevant for alcohol dependence, DCUK-OEt should be further evaluated for the treatment of alcoholism.

## Introduction

GABA (γ-aminobutyric acid) is the major inhibitory transmitter and glutamate is the major excitatory transmitter in brain and these two opposing forces are in constant interplay within the communication systems of the brain^1^. The desire for pharmacological manipulation of GABAergic neurotransmission has generated a plethora of xenobiotics which are useful in medicine, including anticonvulsants, anesthetics, anxiolytics, muscle relaxants and medications for treating pain. The realization that the GABA_A_ receptor system is a collage derived from 6 α, 3 β, 3 γ, δ, θ, ε, π and 3 ρ subunits^2, 3^, and that different combinations of these subunits are particularly important in certain physiologic events mediated by GABA, has stimulated a search for chemical entities that have selectivity for GABA_A_ receptors with a particular combination of subunits^4, 5^.

**Figure 1:**
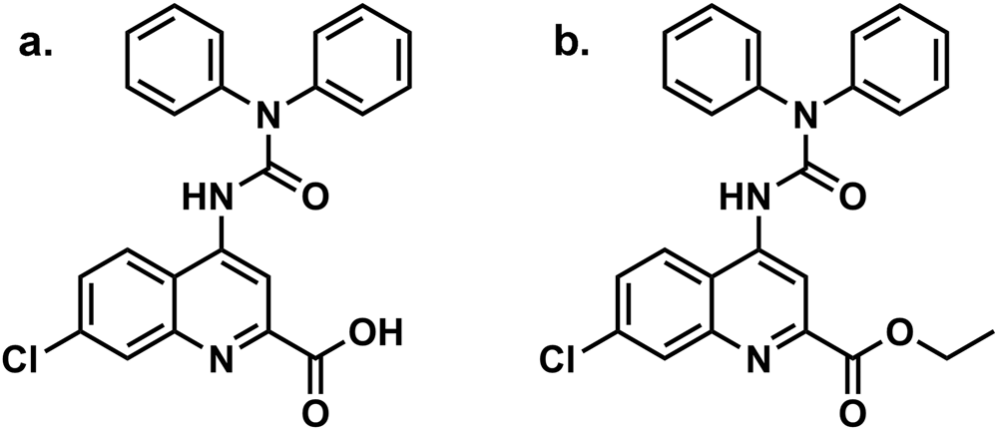
Chemical structure of DCUK compounds. (**a**) DCUKA (5,7-Dichloro-4-([diphenyl carbamoyl] amino) quinoline-2-carboxylic acid). (**b**) DCUK-OEt (5,7-Dichloro-4-([diphenyl carbamoyl] amino) quinoline-2-ethyl carboxylate).

We had previously reported on a “rationally engineered” molecule which effectively reduced allodynia in animal models of neuropathy by simultaneously targeting the NMDA subtype of glutamate receptor and voltage-sensitive sodium channels^6^, particularly Na_v_1.7^7^ and Na_v_1.8^8^. This compound showed neither sedative effects *per se*, nor did it enhance the sedative or motor incoordinating effects of ethanol. We more recently generated a number of chemical derivatives of the “skeleton quinoline structure” of our original molecule. In screening these molecules through a series of radioligand binding assays^9^ we found that 5,7-dichloro-4-([diphenyl carbamoyl] amino) quinoline-2-ethyl carboxylate (DCUK-OEt) (Fig. 1) could displace muscimol from its specific binding sites in an assay containing washed rat brain membranes, while it had no effect at a concentration of 10 μM in 32 other radioligand binding assays. The current manuscript describes the equilibrium radioligand binding studies and electrophysiological analysis of the effects of DCUK-OEt, as well as the non-esterified derivative, 5,7 dichloro-4-([diphenyl carbamoyl] amino) quinoline-2-carboxylic acid (DCUKA) (Fig. 1) which is the primary metabolite of DCUK-OEt, on GABA_A_ receptors. The electrophysiological studies were carried out in *Xenopus laevis* oocytes and in neurons from the rat central amygdala (CeA). The GABA_A_ subunit combinations tested in oocytes were selected based on their abundance in brain (e.g. α1β2γ2) and their expression in the CeA^10–14^. Additional subunits were expressed with the objective of further elucidating the selectivity of the DCUK compounds. The results indicate that DCUK-OEt may have characteristics which distinguish it from all currently available ligands that act on the GABA_A_ receptor.

**Table 1:** Displacement of Ligands Binding to GABA_A_ Receptors by DCUK-OEt and DCUKA.

Compound	[^3^H]Muscimol Binding	[^3^H]Flunitrazepam Binding
DCUK-OEt	1.7 ± 0.3 μM	>10 μM
DCUKA	6.6 ± 1.9 μM	>10 μM

IC_50_ and Ki values were obtained by non-linear regression analysis of radioligand binding isotherms. Ki values are reported as estimates from the non-linear regressions and their associated standard errors (n = 10 points in the binding isotherms).

## Results

The radioligand displacement studies that were performed with [^3^H]flunitrazepam and [^3^H]muscimol, utilized washed rat brain membranes and thus represented an amalgam of GABA_A_ receptors composed of various subunit combinations. Neither DCUK-OEt nor DCUKA demonstrated efficacy for displacing [^3^H]flunitrazepam. However, at concentrations <10 μM, both DCUK-OEt and DCUKA were able to displace [^3^H]muscimol, albeit with different potency. The K_i_ for displacement of muscimol binding by DCUKA was 6.6 μM and displacement by DCUK-OEt demonstrated a lower K_i_ of 1.7 μM (Table 1). DCUK-OEt at concentrations <10 μM demonstrated no significant displacement of any of the ligands selective for 32 other receptors/transporters/channels that were tested in the course of our studies (Supplementary Table S1).

**Figure 2:**
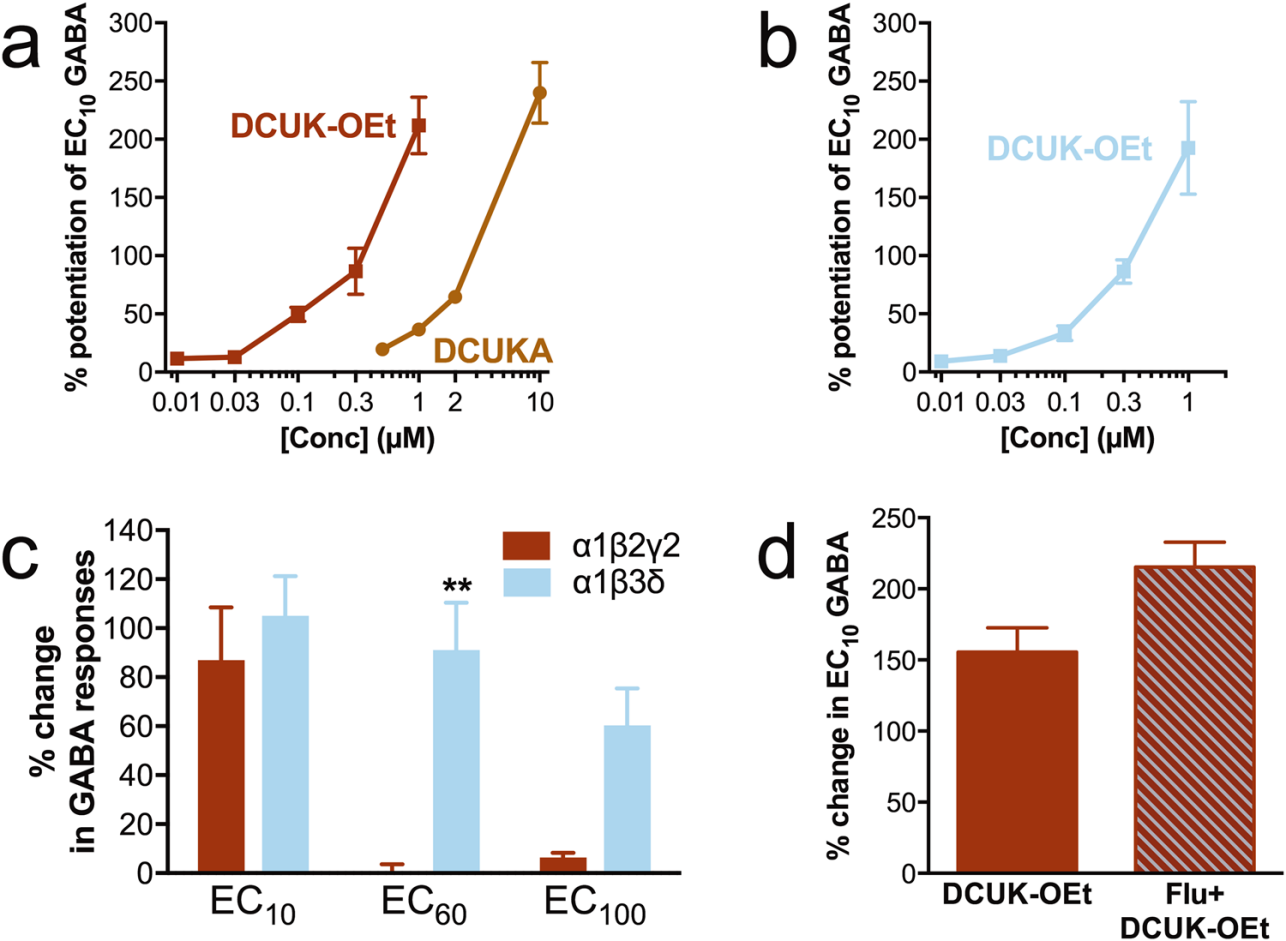
DCUK effect on GABA responses. (**a**) Effect of DCUK compounds on submaximal (EC_10_) GABA responses of α1β2γ2 GABA_A_ receptors (n = 4–5 at each concentration of DCUK compound). (**b**) Effect of DCUK-OEt on submaximal (EC_10_) GABA responses of α1β3δ GABA_A_ receptors (n = 5–6 at each concentration of DCUK-OEt). (**c**) Effect of DCUK-OEt (0.3 µM) and escalating GABA concentrations applied to α1β2γ2 and α1β3δ GABA_A_ receptors (n = 9 each). GABA concentrations used: 3 and 1 µM for α1β2γ2 and α1β3δ, respectively (~EC_10_); 30 µM (~EC_60_); 3 mM (~EC_100_). (**d**) DCUK-OEt (0.3 μM) effect in the absence and presence of 20 μM flumazenil (Flu) (n = 5 for each condition). Data represent mean ± SEM. **p < 0.01 compared to α1β2γ2 (one-way ANOVA and post-hoc contrasts).

Both DCUK-OEt and DCUKA enhanced submaximal GABA (EC_10_) currents in oocytes containing α1β2γ2 GABA_A_ receptors (Fig. 2a). Full concentration-response curves were not possible due to solubility limits, but, from the partial curves, equi-effective concentrations were approximately 10-fold lower for DCUK-OEt than for DCUKA (e.g., 0.3 μM DCUK-OEt had the same effect as 3 μM DCUKA). DCUK-OEt was similarly effective in potentiating submaximal GABA currents in α1β3δ and α1β2γ2 GABA_A_ receptors (Fig. 2a and b). Interestingly, DCUK-OEt potentiated GABA currents produced by higher concentrations of GABA (EC_60_ and EC_100_) with α1β3δ GABA_A_ receptors, but not with α1β2γ2 GABA_A_ receptors (Fig. 2c). Representative tracings of GABA-induced currents in the presence of DCUK-OEt are shown in Supplementary Fig. S1. The positive modulation of GABA_A_ receptors by DCUK-OEt was specific to the GABA_A_ family of heteromeric receptors and even closely related receptors such as ρ1 GABA_A_ and α1 Gly receptors showed no evidence of positive allosteric modulator (PAM) activity with DCUK-OEt (DCUK-OEt produced a small but statistically significant reduction in ρ1 receptor currents, Supplementary Fig. S2).

A prominent group of positive allosteric modulators of GABA_A_ receptors act through the benzodiazepine site, located at the extracellular interface between the α and the γ subunit^4^. To test whether the DCUK compounds acted through this site, we co-applied flumazenil. Flumazenil can act as a partial agonist at the benzodiazepine site, and 20 μM flumazenil alone potentiated EC_10_ GABA responses (51 ± 2%, n = 5). However, flumazenil did not significantly affect either DCUK-OEt (Fig. 2d) or DCUKA (not shown) actions on α1β2γ2 GABA_A_ receptors, while significantly inhibiting flunitrazepam PAM actions. In these studies, 0.1 µM flunitrazepam produced a 108 ± 9% potentiation of the EC_10_ GABA response, but that potentiation was diminished to 34 ± 2% in the presence of 20 µM flumazenil.

The neurosteroids are another group of allosteric modulators of GABA_A_ receptors when applied at low concentrations. We used a partial antagonist of 5α-reduced neurosteroids [17PA, 17-phenyl-(3α,5α)-androst-16-en-3-ol)]^15^ to test whether DCUK-OEt acts through this site. When coapplied with DCUK-OEt, 17PA produced 35% inhibition of DCUK-OEt potentiation of GABA actions, while it inhibited 56% of the potentiating effects of allopregnanolone on GABA-induced currents (Supplementary Fig. S3).

The composition of the GABA_A_ receptors was critical in determining the effects of DCUK-OEt. When applied to α1β2, α5β3 or α1β3 GABA_A_ receptors, the average effect of 0.3 μM DCUK-OEt was not significantly different from zero (Table 2). A third subunit (either γ or δ) definitively increased the DCUK-OEt PAM effect, and the identity of the third subunit was quite relevant to the magnitude of the PAM effect. For instance, DCUK-OEt induced less potentiation (non-significant) of the GABA responses with α1β2γ1 GABA_A_ receptors compared with α1β2γ2 GABA_A_ receptors (Tables 2 and 3).

**Table 2:** DCUK-OEt (0.3 μM) induced change in the response to EC_10_ GABA in GABA_A_ receptors composed of different subunit combinations.

Receptor	Percent Change	Standard Error	Sample Size	Unadjusted p-value	Bonferroni adjusted p-value	Effect
α1β2	40	**19**	11	0.035	0.42	Non-significant
α1β3	20	**27**	5	0.449	>0.99	Non-significant
α5β3	8	**25**	8	0.730	>0.99	Non-significant
α1β1γ2	17	**23**	11	0.445	>0.99	Non-significant
α1β2γ2	127	**10**	44	<0.001	<0.01	Significant
α1β2(N265S)γ2	45	**18**	14	0.013	0.16	Marginal
α1β2γ1	56	**24**	8	0.022	0.26	Non-significant
α1β3γ2	95	**29**	6	0.002	0.02	Significant
α4β3γ2	49	**26**	8	0.066	0.79	Non-significant
α5β3γ2	81	**24**	8	0.001	0.01	Significant
α1β3δ	102	**16**	18	<0.001	<0.01	Significant
α4β3δ	−6	**31**	5	0.842	>0.99	Non-significant

Significant and marginal effects are those with a Bonferroni-adjusted p-value < 0.05 and < 0.2, respectively. A linear mixed model was implemented in SAS (version 9.4) to calculate the normalized percent change in current for each receptor subunit combination produced by DCUK-OEt (EC_10_ GABA concentration without and with 0.3 μM DCUK-OEt). A random effect of batch was included in the model, and for each receptor, the percent change in the GABA-induced current produced by DCUK-OEt was compared to 0 using a single-sample t-test in the MIXED procedure in SAS and a Bonferroni adjustment to correct for multiple comparisons.

**Table 3:** Comparison of DCUK-OEt induced changes in EC_10_ GABA responses between receptors differing in a single subunit.

Receptor 1	Receptor 2	Percent Difference (Receptor 1–2)	Standard Error	Unadjusted p-value	Bonferroni adjusted p-value	Effect
α1β2	α1β2γ2	−87	19.1	<0.01	<0.01	Significant
α1β2γ2	α1β1γ2	110	24.7	<0.01	<0.01	Significant
α1β2γ2	α1β3γ2	32	30.9	0.30	>0.99	Non-significant
α1β2γ2	α1β2(N265S)γ2	82	17.5	<0.01	<0.01	Significant
α1β1γ2	α1β2(N265S)γ2	−28	28.7	0.34	>0.99	Non-significant
α1β1γ2	α1β3γ2	−77	36.9	0.04	0.69	Non-significant
α1β2γ2	α1β2γ1	71	24.6	<0.01	0.09	Marginal
α1β2	α1β3	20	32.5	0.54	>0.99	Non-significant
α1β3	α5β3	12	36.3	0.74	>0.99	Non-significant
α1β3	α1β3γ2	−74	39.6	0.06	>0.99	Non-significant
α5β3	α5β3γ2	−72	34.5	0.04	0.69	Non-significant
α1β3γ2	α4β3γ2	46	39.2	0.24	>0.99	Non-significant
α1β3γ2	α5β3γ2	14	38.0	0.71	>0.99	Non-significant
α4β3γ2	α5β3γ2	−32	35.7	0.37	>0.99	Non-significant
α1β3	α1β3δ	−82	27.9	<0.01	0.07	Marginal
α1β3γ2	α1β3δ	−8	33.2	0.82	>0.99	Non-significant
α4β3γ2	α4β3δ	55	36.8	0.14	>0.99	Non-significant
α1β3δ	α4β3δ	109	35.0	<0.01	0.05	Significant

These comparisons were executed with the linear mixed model using linear contrasts. Correction for multiple pairwise comparisons was by a Bonferroni adjustment.

The identity of the α subunit also contributed to the magnitude of the DCUK-OEt effect: DCUK-OEt significantly potentiated GABA responses of α1β3δ, but not α4β3δ GABA_A_ receptors (Tables 2 and 3). DCUK-OEt similarly potentiated α1β3γ2 and α5β3γ2 GABA_A_ receptors, but the PAM effect was not significantly different from zero for α4β3γ2 GABA_A_ receptors (Table 2). The identity of the β subunit also played a role in the magnitude of the DCUK-OEt effect as a PAM: the α1β1γ2 GABA_A_ receptors showed no significant potentiation of the GABA responses by DCUK-OEt while α1β2γ2 and α1β3γ2 GABA_A_ receptors did (Table 2). The β1 subunit residue 265 seems to play an important role in determining the effect of certain GABA_A_ receptor modulators: when S265 in β1 is mutated to N (homologous residue in β2 and β3) on the GABA_A_ receptor complex, the modulators’ potentiation is increased, and vice versa, when N265 in β2 or β3 is mutated to S, the potentiation is reduced^16–18^. When we tested DCUK-OEt on α1β2(N265S)γ2 compared to α1β2γ2, the effect of DCUK-OEt as a PAM was significantly reduced (Table 3), but not to the extent seen with drugs such as etomidate (no GABA potentiating effect of etomidate at concentrations up to 1 mM was evident with the α1β2(N265S)γ2 receptor combination)^16^.

**Figure 3:**
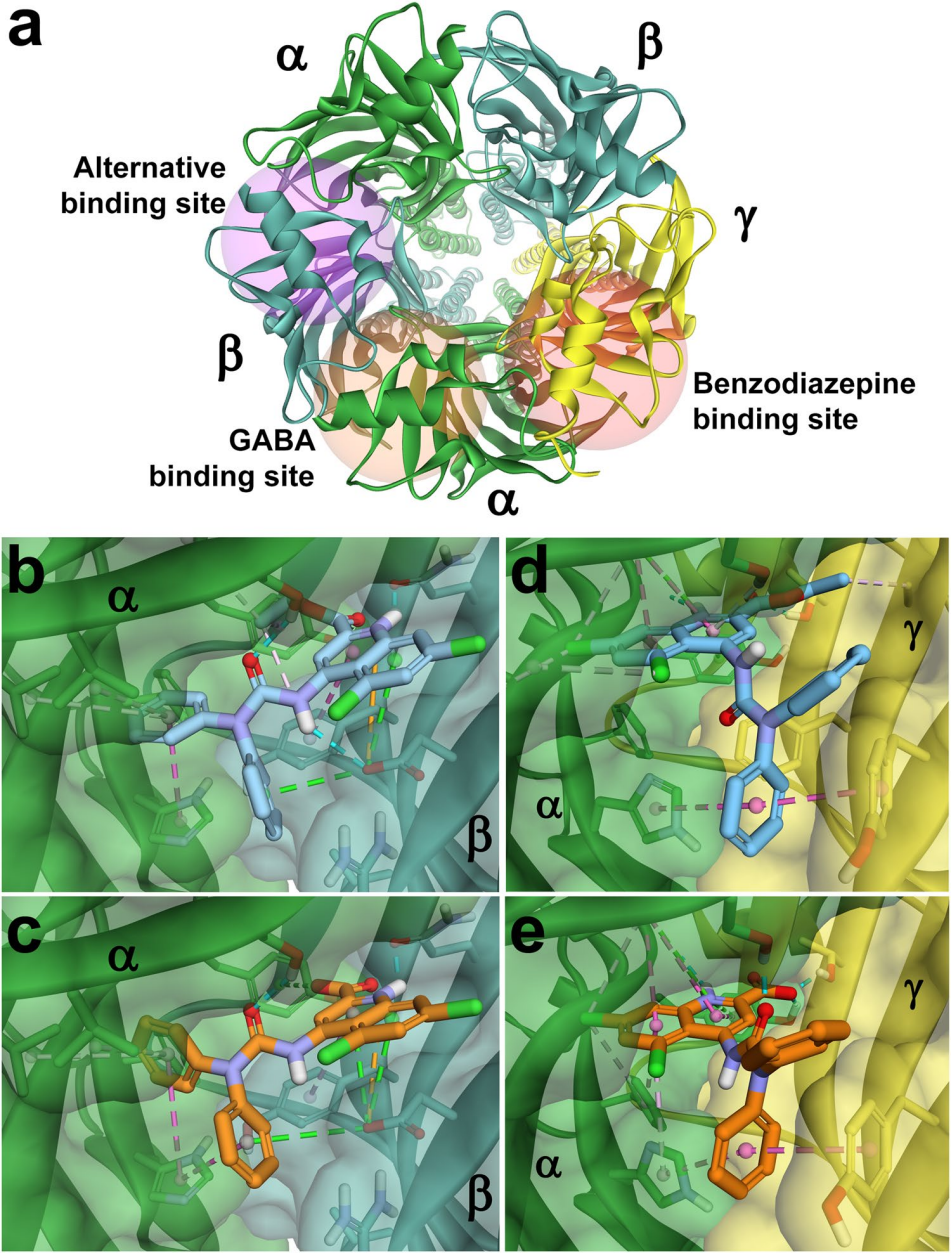
Predicted docking of DCUK-OEt and DCUKA within extracellular domain interfaces of GABA_A_ receptor subunits. The α subunit is shaded in green, β in cyan and γ in yellow. (**a**) Extracellular (top down) view of the pentameric GABA_A_ receptor. The interfaces illustrated are α + β- (alternative site), and α + γ- (benzodiazepine site). (**b**) DCUK-OEt and (**c**) DCUKA within the alternative site (α + β-). DCUK-OEt is represented by orange sticks and DCUKA is represented by pink sticks. (**d**) DCUKA and (**e**) DCUK-OEt within the benzodiazepine site (α + γ-). Dashed lines indicate predicted non-bond interactions (green = H-bonds, orange = electrostatic or π-cation/anion, magenta = π-π, purple = π-σ, pink = hydrophobic).

To further investigate potential binding sites for DCUK-OEt on the GABA_A_ receptor, we performed computationally-based small molecule docking studies to compare the potential interactions of DCUK-OEt with those of DCUKA, flunitrazepam, and etomidate, with either the classical benzodiazepine binding site (located at the α + γ- interface of the pentameric receptor) or an alternative binding site (at the α + β- interface) (Fig. 3a). The corresponding binding energies are shown in Table 4. These studies indicated that DCUK-OEt exhibited the highest predicted affinity for an alternative binding site, while, as expected, flunitrazepam exhibited the highest predicted affinity for the benzodiazepine site.

**Table 4:** Docking binding energies and interactions at GABA_A_ receptor sites.

**Compound**	**Binding energy**	**H-bonds/Electrostatic**	**π-π**	**π-Anion/Cation**	**π-σ**	**Hydrophobic**	
**Alternative site (α + β- interface)**							
DCUK-OEt	−55.9	αTyr160, αSer205; βAsp43, βGln64	αHis102; βTyr62	βAsp43	αTyr160	αVal203, αTyr210, αVal212; βTyr62	
DCUKA	−40.1	αTyr160, αSer205; βGln64	αHis102	βAsp43	—	αVal203, αVal212	
Flunitrazepam	−33.7	βAsp43, βArg180	βTyr62	—	—	αTyr210	
Etomidate	−20.7	—	βTyr62	βAsp43	—	αPhe100, αTyr160; βTyr62	
**Compound**	**Binding energy**	**H-bonds/Electrostatic**	**π-π**	**π-Anion/Cation**	**π-σ**	**Hydrophobic**	**π-Amide**
**Benzodiazepine site (α + γ- interface)**							
DCUK-OEt	−47.8	αTyr160; γThr142	αHis102, αTyr210; γTyr58	αTyr160, αTyr210	—	αVal203, αTyr210, αVal212; γAla79	—
DCUKA	−59.7	αTyr160 (x2), αSer205; γThr142	αPhe100, αHis102, αTyr160, αTyr210; γTyr58	αTyr160, αTyr210	—	αPhe100, αHis102, αTyr210	—
Flunitrazepam	−79.5	αTyr160; γThr142	αHis102, αTyr160, αTyr210; γTyr58, γPhe77	αTyr160	αPhe100	—	—
Etomidate	−16.3	—	γTyr58	—	—	αHis102, αVal203	αGln204, αSer205

Summary of the binding energies and non-bond interactions of the top scoring predicted binding orientations for each compound docked into the homology model of the benzodiazepine binding site at the α + γ- subunit interface or the “Alternative” binding site at the α + β- subunit interface of the human GABA_A_ receptor shown in Fig. 2 and in Supplementary Fig. S4. Binding orientations were predicted using the Discovery Studio flexible docking protocol and energies were calculated using the distance-dependent dielectric model, as outlined in the methods.

The modeling studies predicted both the carboxylate of DCUKA and the ethyl ester moiety of DCUK-OEt to be oriented towards the α subunit in the region of α:Tyr160 in the alternative site (Fig. 3b and c). The ethyl ester was predicted to participate in additional hydrophobic interactions with the residues of this region, and there exists a potential π-σ interaction with α:Tyr160. These additional interactions of the ethyl ester also appeared to optimize the positioning of the head group and amide linker within the binding pocket to allow for additional potential H-bond and π-π interactions with β:Asp43 and Tyr62, respectively, leading to the higher affinity for DCUK-OEt compared to DCUKA for the GABA_A_ receptor.

The predicted binding and interactions of flunitrazepam in the benzodiazepine site (Supplementary Fig. S4) were consistent with previous studies^19–21^, and flunitrazepam made a number of favorable contacts, including H-bond interactions with α:Tyr160 and γ:Thr142, and π-π interactions with α:Tyr160 and Tyr210. DCUKA shared a number of these predicted contacts, while the ethyl ester of DCUK-OEt appeared to impair the optimal positioning of the head group in the benzodiazepine binding pocket (Fig. 3d and e). Flunitrazepam bound somewhat more deeply into the pocket, compared to the other tested compounds, with the fluorbenzene ring predicted to be locked in place by a three-way π-stacking interaction with α:His102 and γ:Tyr58, Phe77. An additional π-σ interaction with α:Phe100 and π-π stacking with γ:Phe77 not only distinguish the predicted binding of flunitrazepam from DCUKA, but also represent the crucial aspects of interaction of flunitrazepam with the receptor that lead to its pharmacological function. The presence of the phenyl (C ring) substitution at the 5 position of the benzodiazepine ring structure is necessary for the PAM actions of the benzodiazepine derivatives^22, 23^. Therefore, even though DCUKA and DCUK-OEt may bind to the benzodiazepine binding site on the GABA_A_ receptor (with lower affinity), the lack of the fluorbenzene ring on the DCUKA and DCUK-OEt structures would predict the lack of functional effect of DCUKA and DCUK-OEt via the benzodiazepine site. It is important to note that, due to the method by which binding energies are calculated, comparisons of relative binding affinity can only be reliably assessed between different molecules within the same binding site.

The studies showing that potentiation of GABA responses by DCUK-OEt cannot be blocked by flumazenil do not preclude the possibility, suggested by the docking experiments, that DCUK-OEt could bind to the benzodiazepine site as an antagonist, while producing potentiation via binding to a different site (the alternative, extracellular site or a transmembrane one). We tested this hypothesis by co-applying DCUK-OEt (1 µM) and flunitrazepam (0.1 µM). The combined effect was larger than the sum of their individual effects (Supplementary Fig. S5), suggesting that the functional effects of the two drugs may be mediated by actions at two different sites.

**Figure 4:**
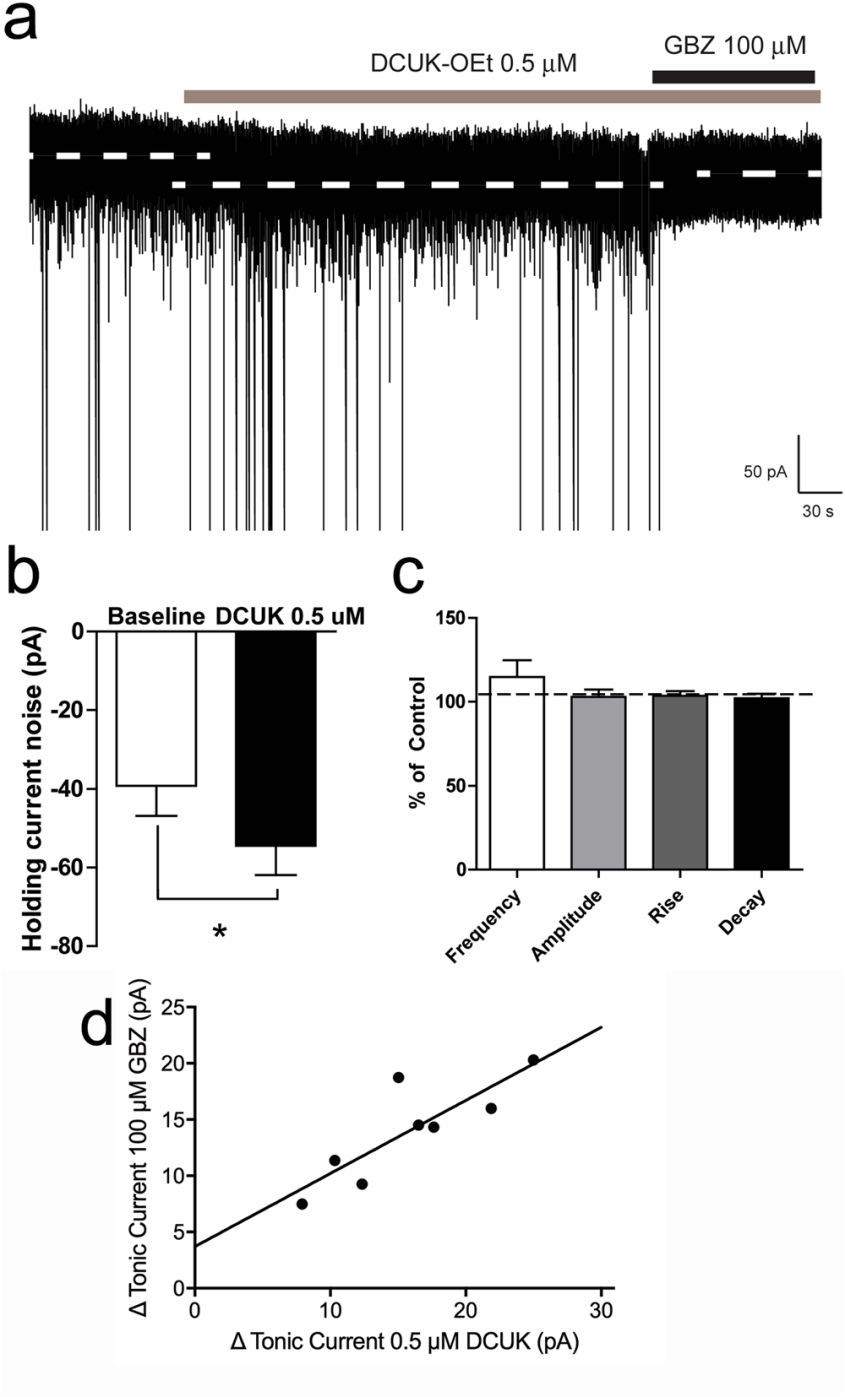
DCUK-OEt potentiates tonic currents in medial CeA neurons. (**a** and **b**) Focal application of DCUK-OEt (0.5 µM) significantly increased the holding current in medial CeA neurons (*p < 0.05, paired t-test). (**c**) No change was evident in frequency, amplitude, rise and decay of mIPSPs with focal application of DCUK-OEt. (**d**) Correlation of magnitude of increase in tonic current produced by 0.5 μM DCUK-OEt with reduction of current by subsequent application of 100 μM gabazine. To demonstrate that changes in holding current were due to increases in tonic signaling, the GABA_A_ receptor antagonist gabazine (GBZ) (100 μM) was focally applied following DCUK-OEt application. For all graphs, n = 11 cells.

The significant effects of DCUK-OEt on particular subunit combinations of the GABA_A_ receptor led us to test the effects of this compound on neurons in the rat central amygdala (CeA). The CeA is primarily composed of GABAergic neurons and changes in CeA GABAergic neurotransmission have been implicated in the development and maintenance of alcohol dependence^24^. Focal application of DCUK-OEt (0.5 µM) significantly increased the holding current in medial CeA neurons (Fig. 4a and b), while producing no significant effect on spontaneous inhibitory postsynaptic current (sIPSC) frequency, amplitude, rise or decay times (Fig. 4c).

To confirm that the changes in holding current that we observed were due to increases in tonic signaling at the GABA_A_ receptor, the GABA_A_ receptor antagonist gabazine (GBZ, 100 μM, Sigma Chemical Co., St. Louis, MO) was focally applied following DCUK-OEt application. GBZ produced a significant reduction in holding current when applied after DCUK-OEt, suggesting that the changes in holding current that were observed with DCUK-OEt, were due to DCUK-OEt-induced increases in tonic conductance via GABA_A_ receptors on medial CeA neurons. In addition, we found that the increase in holding current with DCUK-OEt was positively correlated with the reduction in holding current seen with GBZ application (Pearson correlation coefficient = 0.838; p = 0.0094; n = 8; Fig. 4d).

## Discussion

DCUK-OEt acts as a subunit-selective PAM at the GABA_A_ receptor, and our ligand binding studies produced no evidence of interaction of DCUK-OEt (<10 μM) with 32 other receptors/transporters/channel proteins. DCUK-OEt exhibited its most robust effects on submaximal GABA-induced currents when applied to the α1β2γ2 GABA_A_ receptor, the subunit combination most highly expressed in mammalian brain^2^. Similar PAM activity of DCUK-OEt was exhibited with GABA_A_ receptors composed of α1β3δ subunits. On the other hand, DCUKA, which lacks the ester moiety at the 2 position of the carboxyquinoline, and is the major metabolite of DCUK-OEt, was 10 times less potent than DCUK-OEt in acting as a PAM at the α1β2γ2-containing GABA_A_ receptors.

The most studied PAMs at the GABA_A_ receptor are benzodiazepine derivatives and other compounds (e.g., zolpidem) which act at the interface of extracellular domains of the α and γ subunits^4^. Our data produced no evidence for DCUK-OEt action at this site. DCUK-OEt did not displace flunitrazepam in ligand displacement experiments, and the electrophysiological effects of DCUK-OEt (and DCUKA) on GABA_A_ receptors expressed in oocytes were not modified by the selective benzodiazepine antagonist, flumazenil. Additionally, the substitution of a δ subunit for a γ subunit in the GABA_A_ receptor complex greatly diminishes the effects of benzodiazepines^25^ but the effects of DCUK-OEt were similar in receptors containing either γ2 or δ subunits (compare α1β3γ2 and α1β3δ in Tables 2 and 3). Finally, when DCUK-OEt and flunitrazepam were applied together, the PAM effect was supra-additive.

Assessment of the possibility that DCUK-OEt acted at the “neurosteroid” site on the GABA_A_ receptor produced somewhat equivocal results. 17PA, which has been reported^15, 26^, and in our hands also shown, to be a weak partial antagonist at the “neurosteroid” site on the GABA_A_ receptor, produced a statistically significant but modest (35%) inhibition of the PAM actions of DCUK-OEt, while inhibiting the effects of allopregnanolone by 56%. This difference in potency of 17PA could be due to differences in the affinity/efficacy of DCUK-OEt compared to allopregnanolone at the “neurosteroid” site(s). However, neurosteroid agonists acting at a “neurosteroid” site^26^ are particularly effective as PAMs, and are also direct agonists at GABA_A_ receptors composed of the α4βxδ subunits, while DCUK-OEt had no significant effect on this subunit combination. Furthermore, the modulatory action of neurosteroids at low concentrations does not differ among β subunits^27^. On the other hand, both β2 and β3 subunits in combination with α1 and γ2 subunits responded to the addition of DCUK-OEt with a significant increase in the current induced by submaximal GABA, but the substitution of the β1 subunit for either β2 or β3 resulted in a notable decrease of the PAM activity of DCUK-OEt (Table 2). The negative effect of the β1 subunit is reminiscent of the selectivity for β subunits shown by modulators such as loreclezole^18^ and etomidate^28, 29^, among others. Three amino acids in the transmembrane domains of the β subunit, distinguish the sequence of β1 from β2/β3^30^, and mutation of the asparagine at position 265 in the β2 sequence, located at the interface of α/β transmembrane domains, has been demonstrated to interfere with the potentiating action of etomidate and other anesthetics at GABA_A_ receptors^16, 17, 30, 31^. The introduction of a mutated β2 (N265S) into a complex containing α1 and γ2 subunits significantly reduced (Table 3) the PAM activity of DCUK-OEt. However, this mutation has been shown to eliminate etomidate’s PAM action^28, 32^. Mutation of β2N265 also decreases alcohol PAM activity on GABA_A_ receptors^33, 34^. However, ethanol potentiates GABA effects at receptors composed of dimeric αβ GABA_A_ receptors, and does not discriminate between β1 versus β2 subunits^35^. Reports on the concentrations of ethanol necessary to potentiate the effects of GABA on α4β3δ GABA_A_ receptors expressed in *Xenopus* oocytes have been contradictory^36–38^, but the ethanol effect on the α4β3δ subunit combination is always potentiation of the GABA actions, in contrast to the lack of any significant effect of DCUK-OEt.

At the EC_10_ concentration of GABA, DCUK-OEt exhibited PAM effects on α1β3δ GABA_A_ receptors similar to effects seen with α1β2γ2. However, DCUK-OEt also enhanced the current produced by saturating concentrations of GABA with the α1β2/3δ subunit combination, but not with the α1β2/3γ2 combination (Fig. 2c). GABA has been shown to be a partial agonist at δ subunit-containing receptors^39^, and DCUK-OEt, and some other PAMs^40^, may allow for further activation of the GABA_A_ receptor at concentrations seemingly maximal in the absence of PAMs. It also should be stressed that we detected no effect of DCUK-OEt at any concentration on any of the subunit combinations we tested in our paradigm, without the addition of GABA.

Overall, as noted above, there seems to be some overlap in the characteristics of DCUK-OEt with properties exhibited by allopregnanolone, CGS 9895, LAU-177^41, 42^, loreclezole, etomidate and ethanol, but other characteristics regarding subunit selectivity of DCUK-OEt mitigate against assuming that DCUK-OEt binding/activity occurs specifically through the currently described site(s) for binding of these agents. Additionally, DCUK-OEt characteristics do not conform to what would be expected if DCUK-OEt were utilizing the canonical barbiturate, or intravenous or inhalation anesthetic sites to affect GABA action at the GABA_A_ receptor^31, 43–45^.

Our models to ascertain the docking of DCUK-OEt to interfaces between the various subunits of the GABA_A_ receptor (composed of α1β2γ2 subunits), indicated that a binding site for DCUK-OEt may exist between the α + β- interface in the pentameric receptor. The free energy (−ΔG) of binding at this site was highest for DCUK-OEt and lowest for etomidate and flunitrazepam. When examining the docking at the benzodiazepine site located between the α + γ- interface, the order was reversed, with flunitrazepam showing the highest binding energy and DCUK-OEt and etomidate showing the lowest −ΔG. If the function of DCUK-OEt was dependent on binding at a single site at the α + β- interface, one would expect that GABA_A_ receptors composed of only α and β subunits would respond as well as the receptors which also contain the γ or δ subunit. This was not the case, and the presence of the γ or δ subunit was necessary to exhibit the PAM action of DCUK-OEt. In fact, the type of γ subunit expressed with the α and β subunits was important, with the γ1 subunit being significantly less effective than the γ2 subunit. Because of the absence of the phenyl ring substituent (C ring) that generates functional (PAM) benzodiazepine derivatives, DCUK-OEt would not be expected to be an agonist at the benzodiazepine site, and our electrophysiologic experiments in the presence of flumazenil support this contention. It was, however, interesting that the combined effects of flunitrazepam and DCUK-OEt produced significantly more than an additive effect, possibly indicating an allosteric interaction between the benzodiazepine site and the site on the α + β- interface which binds DCUK-OEt with higher affinity.

The radioligand binding studies that led us to the electrophysiological examination of DCUK-OEt on the GABA_A_ receptor, also produced some insight into the possible mechanism by which DCUK-OEt may generate its effects. DCUK-OEt produced a decrease in the affinity for muscimol at the GABA_A_ receptor. Such action may be expected if DCUK-OEt is shifting the GABA_A_ receptor into a state more likely to be in an open channel configuration. The GABA_A_ receptor has been shown to display two affinity states for agonists such as muscimol^46, 47^ and the high affinity state of the GABA_A_ receptor has been proposed to represent stabilization of the desensitized form of the receptor^48^. One can speculate that DCUK-OEt is increasing the proportion of receptors in a low affinity state at any particular concentration of agonist (muscimol). This speculation will require more investigation, but it is interesting that ethanol^49^ and the anxiolytic/anticonvulsant etifoxine^50^, which both can act as PAMs at lower concentrations, reduce muscimol affinity at GABA_A_ receptor in rat brain membrane preparations.

The α1β2γ2 combination of subunits is the primary combination of synaptically localized GABA_A_ receptors in brain that mediate phasic inhibition, while α1/4/6βxδ receptors have been considered to be the primary type of extrasynaptic GABA_A_ receptors that mediate tonic inhibition^51^. Given our results with GABA_A_ receptors containing α4 and α1 subunits together with the γ2 or δ subunit, one could assume that DCUK-OEt would well affect the function of synaptically localized GABA_A_ receptors as well as certain extrasynaptic GABA_A_ receptors. We noted two characteristics of DCUK-OEt that suggest that its primary effect may be at extrasynaptic receptors containing either a γ2 or δ subunit together with an α1 and β3 subunit. These combinations of subunits (α1β3γ2 and α1β3δ) display a low EC_50_ for GABA (see Supplementary Fig. S6) and DCUK-OEt can produce highly significant potentiation of α1β3γ2 and α1β3δ-mediated currents at the EC_10_ concentration of GABA in our assays, and probably at concentrations of GABA consistent with those encountered in locations outside of the GABA synapse. This observation would be quite compatible with significant potentiation at extrasynaptic sites where concentrations of GABA have been considered to be in the high nM range, as opposed to the high concentrations (mM) of GABA that are present in the synapse^52^. We saw no measurable effect of DCUK-OEt on α1β2γ2 receptors at high concentrations of GABA (EC_60_ and above), and non-significant effects on α1β1γ2 and α1β2γ1 GABA_A_ receptors at low GABA concentrations (EC_10_). Since αβγ is responsible for the major portion of the phasic actions of GABA, and relatively high amounts of β1 and γ1 were reported at synaptic sites in CeA^10–12, 14^, it is plausible that phasic effects of GABA through these subunit combinations would not be modulated by DCUK-OEt. In fact, when we applied DCUK-OEt focally to CeA neurons, we found no change in sIPSC frequency, amplitude, rise or decay time, indicating no effects on phasic transmission (Fig. 4c).

There is strong evidence for the existence of α1βxδ receptors located extrasynaptically in particular areas of brain (i.e., the interneurons of the hippocampus and particularly those of the dentate gyrus)^53–55^. Tonic inhibition mediated by GABA_A_ receptors containing the α1 subunit has also been noted in the CeA^56^. Our prior studies using slices of the CeA demonstrated that CRF1 receptor-positive (CRF1+) neurons express the α1 GABA_A_ receptor subunit, and this subunit is integral for the GABA-mediated tonic conductance in these neurons as well as being involved in the phasic synaptic response to GABA^56^. When we measured tonic conductance in CeA neurons, focal application of DCUK-OEt produced an enhancement of the recorded tonic current, suggesting local effects of DCUK-OEt at extrasynaptic GABA_A_ receptors. To further ascertain whether the effects of DCUK-OEt were mediated particularly by extrasynaptic GABA_A_ receptors, we performed a comparison of the change (increase) in current produced by DCUK-OEt and the decrease generated by the subsequent co-application of 100 μM gabazine^57^. The strong correlation indicated that DCUK-OEt was indeed stimulating a tonic conductance in these neurons by actions at extrasynaptic GABA_A_ receptors. Recently, de Guglielmo *et al*.^58^ reported that inactivation of an ensemble of neurons in the CeA resulted in abrogation of excessive alcohol consumption by alcohol-dependent rats. The anatomical area of the CeA from which we obtained our electrophysiologic data coincides with the area containing the ensemble described by de Gugielmo *et al*.^58^. An increase in the tonic conductance through extrasynaptic GABA_A_ receptors, mediated by DCUK-OEt, may engender reduced activity of the neurons identified by de Guglielmo *et al*.^58^ and be an effective mode for reducing alcohol intake by dependent animals.

In all, our characterization of DCUK-OEt indicates that this molecule has characteristics that resemble those of etomidate, other anesthetics, ethanol and neurosteroids, but the full profile of DCUK-OEt actions speaks to an interaction with a site or sites on the GABA_A_ receptor that distinguish DCUK-OEt from currently known PAMs and direct agonists acting at GABA receptors.

## Methods

### Radioligand binding

#### [^3^H]Flunitrazepam Binding and Displacement by DCUK-OEt

*Membrane Preparation*. These experiments were performed at the University of Colorado Health Sciences Center, Denver, CO. Experiments were approved by the Institutional Animal Care and Use Committee (IACUC) of the University of Colorado, Denver, and were performed in accordance with the NIH Guide for the Care and Use of Laboratory Animals. Male Sprague-Dawley rats (200–250 g) were maintained in an AAALAC-accredited facility and sacrificed by CO_2_ exposure and decapitation. Brains were removed, and membranes were prepared from the forebrain as described previously^6^.

*Ligand binding assay*. The binding of [^3^H]flunitrazepam was assayed in triplicate, using final incubation volumes of 0.55 ml consisting of protein (approx 200–300 mg/ml), [^3^H]flunitrazepam (New England Nuclear) at a concentration of 1 nM, 10 µM GABA and DCUKA or DCUK-OEt at 0, 5, 10, 20, 50, 100 and 200 µM in DMSO solution (final DMSO concentration 0.2%). Nonspecific binding was measured in the presence of 10 µM diazepam. Binding was initiated by addition of protein, followed by incubation at 4 °C for 30 min. Bound and free ligand were separated by rapid filtration under vacuum over Whatman GF/B filters presoaked in buffer in a 24 port Brandel Cell Harvester. Filters were washed with 2 × 5 ml of ice-cold HEPES buffer and dried prior to measurement of bound radioactivity by scintillation counting (Beckman LS3800 scintillation counter) using Ultima Gold XR scintillation cocktail.

#### Displacement of [^3^H]muscimol binding by DCUK-OEt or DCUKA

The assays of [^3^H]muscimol binding were performed by the Psychoactive Drug Screening Program/NIMH (PDSP). Rat brain membranes were prepared as described in the Protocol Manual on the PDSP website (https://pdspdb.unc.edu/pdspWeb/). DCUK-OEt or DCUKA were dissolved in 1.0% v/v DMSO and assayed at 11 concentrations ranging from 0.05 nM to 10 μM (final DMSO concentration, 0.2%). The final concentration of [^3^H]muscimol in the assay mixture was 5 nM. Displacement of [^3^H] muscimol by GABA at concentrations ranging from 10 nM to 10 μM was measured to provide a positive control.

#### Screening for binding of DCUK-OEt to other receptors/transporters/enzymes

Additional ligand binding studies (Supplementary Table 1), were also performed by PDSP and in our laboratories (batrachotoxin binding)^6^. The experimental details for all of the PDSP binding studies can be obtained by connecting to the PDSP website (https://pdspdb.unc.edu/pdspWeb/) and clicking on “Assays” (binding or functional) on the menu bar. PDSP initially performed ligand displacement studies at a default concentration of 10 μM DCUK-OEt. For any receptor/transporter at which the compound generated a 50% or greater displacement of the receptor/transporter-selective ligand, a secondary binding assay was performed to calculate K_i_ values (see below).

*Analysis of ligand binding data*. Specific binding of [^3^H]muscimol or [^3^H]flunitrazepam in the presence of each concentration of DCUK-OEt or DCUKA was calculated by subtracting the nonspecific binding from the total binding and averaging the replicate values. The percentage displacement was calculated by dividing the specific binding in the presence of DCUKA or DCUK-OEt by the specific binding in the absence of DCUKA or DCUK-OEt. SigmaPlot 5.0 graphing software (flunitrazepam binding) or GraphPad Prism 4.0 software (muscimol binding) were used to perform non-linear regression of radioligand binding isotherms. Ki values for DCUK-OEt and DCUKA were calculated from best-fit IC_50_ values by the Cheng-Prusoff method^59^.

### Oocyte electrophysiology

*Xenopus laevis* frogs were obtained from Nasco (Fort Atkinson, WI, USA). All surgery was performed in accordance with a protocol approved by the University of Texas, Austin IACUC and the NIH Guide for the Care and Use of Laboratory Animals. The complementary DNAs encoding the GABA_A_ subunits rat α1, β1, β3, γ2 s, δ, and human β2 were provided by Drs Myles H. Akabas, Paul J. Whiting and Richard W. Olsen. Human γ1 cDNA was synthesized *de novo*, optimized for *Xenopus laevis* oocyte expression and subcloned in pGEMHE by GenScript (Piscataway, NJ). The *in vitro* transcription of GABA_A_ subunits was performed using mMessage mMachine (Life Technologies, Grand Island, NY). After isolation of *Xenopus laevis* oocytes, they were injected with capped complementary RNAs encoding wild-type or mutant subunits in different ratios, depending on the subunits: α1β2γ2 s, 2:2:20 ng; α1β2γ1, 2:2:6 ng; α1β2, 3:3 ng; α1β1γ2, 0.5:0.5:5 ng; α1β3γ2, 0.1:0.1:1 ng; α1β3, 0.5:0.5 ng; α1β3δ, α4β3γ2 and α4β3δ, 0.4:0.4:4 ng.

#### Electrophysiology

The injected oocytes were incubated at 15°C in sterilized Modified Barth’s solution for 1–7 days before recording, and the responses of GABA_A_ receptors expressed in oocytes were studied using two-electrode voltage clamp as described earlier^33, 60^. Oocytes were discarded if the maximal current was over 20 µA or if the baseline was unstable or drifted to positive values.

#### Recording protocols

*GABA concentration*-*response curves*. Increasing concentrations of GABA were applied for 20–30 s (0.1–1000 μM) followed by 5–15 minutes washout. Responses were expressed as percentages of the maximal current (Supplementary Fig. S6).

*Modulator application*. DCUK-OEt and DCUKA stock solutions were prepared in DMSO weekly, then sonicated for 15 min, and stored at 4 °C, protected from light. On the day of the experiment, dilutions were prepared, sonicated for 5 min, and used immediately. The final DMSO concentration in the buffer bathing the oocyte was ≤0.1%. In order to test the effects of DCUKs, the agents were first pre-applied for 1 min and then co-applied with GABA. To verify the presence of a third subunit in expressed subunit combinations, the responses to GABA in the presence of Zn++ (1, 10 or 100 µM) were evaluated (Supplementary Table S2). The application sequence in each instance was as follows: Maximal GABA (20 s application, 15 min washout), EC_10_ GABA (30 s application, 5 min washout), EC_10_ GABA, pre-application of the drug followed by a co-application with EC_10_ GABA, EC_10_ GABA, pre-application of Zn++ immediately followed by a co-application with EC_10_ GABA, EC_10_ GABA. In most cases, we limited to one DCUK application per oocyte. Flumazenil and 17PA were pre-applied before their co-application with GABA. When co-applying with DCUK, the antagonist and DCUK were pre-applied together before their co-application with GABA. Flunitrazepam was not pre-applied before co-application with GABA.

#### Statistical Analysis

Responses to DCUK-OEt were quantified as the percent change in current between the response to the EC_10_ concentration of GABA and the response to the EC_10_ concentration of GABA in the presence of 0.3 μM concentration of DCUK-OEt. To control for batch effects a linear mixed model was implemented in SAS (version 9.4) to calculate the normalized percent change in current for each receptor subunit combination (each receptor combination was examined in two to nineteen separate experiments). Because each receptor was examined across several experiments, a random effect of batch was included in the model. For each receptor, the estimated percent change in the GABA EC_10_-induced current produced by addition of DCUK-OEt was compared to zero by ascertaining whether zero percent change was outside the confidence interval of the measured values. This was accomplished by using a single sample t-test in the MIXED procedure of SAS, and a Bonferroni adjustment to control for multiple comparisons across receptors. Comparisons between receptors with a single subunit difference were executed within the linear mixed model using linear contrasts. A Bonferroni adjustment was used to control for multiple pairwise comparisons. Significant effects are those with a Bonferroni adjusted p-value < 0.05 and marginal effects are those with a Bonferroni adjusted p-value < 0.2.

Other statistical tests (t-test and ANOVA) were applied as indicated in the corresponding table or figure legend.

The GABA concentration response curves (CRCs) were fitted to the following equation:

$$I/{I}_{MAX}=\frac{1}{1+{10}^{(\mathrm{log}E{C}_{50}-\mathrm{log}[GABA])\times {n}_{H}}}$$

where *I/I*_*MAX*_ is the fraction of the maximally-obtained GABA response, *EC*_*50*_ (effective concentration 50) is the concentration of GABA producing a half-maximal response, [*GABA*] is GABA concentration and *n*_*H*_ is the Hill coefficient.

### Brain slice electrophysiology

#### Brain slice preparation

All procedures were approved by the Scripps Research IACUC and were consistent with the NIH Guide for the Care and Use of Laboratory Animals. Slices were prepared from brains of 5 adult male Wistar rats (250–350 g) as described by Herman *et al*.^56^. A single slice was transferred to a recording chamber mounted on the stage of an upright microscope (Olympus BX50WI).

#### Brain slice electrophysiological recording

Neurons were visualized and whole cell patch clamp recordings were made as previously described^56^. Series resistance was typically <15 MΩ and was continuously monitored with a hyperpolarizing 10 mV pulse. Electrophysiological properties of cells were determined by pClamp 10 Clampex software online during voltage-clamp recording using a 5 mV pulse delivered after breaking into the cell. The resting membrane potential was determined online after breaking into the cell using the zero current (I = 0) recording configuration and the liquid junction potential was included in the determination.

DCUKA and DCUK-OEt were prepared as described for the experiments on oocyte electrophysiology. Other drugs were dissolved in aCSF, and all drugs were applied by Y-tubing application for local perfusion primarily on the neuron of interest. To isolate the inhibitory currents mediated by GABA_A_ receptors, all recordings were performed in the presence of glutamate and GABA_B_ receptor blockers^56^. All voltage clamp recordings were performed in a gap-free acquisition mode with a sampling rate per signal of 10 kHz or a total data throughput equal to 20 kHz (2.29 MB/min) as defined by pClamp 10 Clampex software.

#### Data Analysis

Frequency, amplitude and decay of spontaneous inhibitory postsynaptic currents (sIPSCs) were analyzed and visually confirmed using a semi-automated threshold based mini detection software (Mini Analysis, Synaptosoft Inc.). Averages of sIPSC characteristics were determined from baseline and experimental drug conditions containing a minimum of 60 events (time period of analysis varied as a product of individual event frequency) and decay kinetics were determined using exponential curve fittings and reported as decay time (ms). All detected events were used for event frequency analysis, but superimposed events were eliminated for amplitude and decay kinetic analysis. In voltage clamp recordings, tonic currents were determined using Clampfit 10.2 (Molecular Devices) and a previously-described method^61^. Responses were quantified as the difference in holding current between baseline and experimental conditions. Events were analyzed for independent significance using a one-sample t-test and compared using a two-tailed t-test for independent samples, a paired two-tailed t-test for comparisons made within the same recording, and a one-way ANOVA with a Bonferroni *post hoc* analysis for comparisons made between 3 or more groups. All statistical analysis was performed using Prism 5.02 (GraphPad, San Diego, CA). Data are presented as mean ± SEM. In all cases, p < 0.05 was the criterion for statistical significance.

### Molecular modeling

All molecular modeling studies were conducted using Biovia Discovery Studio 2016 (Biovia Inc., San Diego, CA) and all crystal structure coordinates were downloaded from the protein data bank (www.pdb.org). The homology model of the human GABA_A_ receptor pentamer was generated with the MODELLER protocol^62^ utilizing the crystal structures of the human GABA_A_ receptor β3 homopentamer as a template (PDB ID: 4COF^63^, Uniprot accession: P28742). Homology models of the human α1 (Uniprot accession: P14867) and γ2 (Uniprot accession: P18507) subunits were superimposed over the template, with the crystal structure of two β3 subunits, so that the final pentameric model consisted of two α1, two β3, and one γ2 subunits, arranged in an γβαβα pattern (counterclockwise, as seen from above). The resulting final structures were subjected to energy minimization utilizing the conjugate gradient minimization protocol with a CHARMm forcefield and the Generalized Born implicit solvent model with simple switching (GBSW)^64^. The minimization calculations converged to an RMS gradient of <0.01 kcal/mol. The Flexible Docking protocol^65^, which allows flexibility in both the protein and the ligand during the docking calculations, was used to predict the binding orientations of both known and candidate GABA_A_ PAMs in the binding site located at either the classical α-γ benzodiazepine site (α + γ- interface) or the alternative α-β site (α + β- interface). Predicted binding poses were energy-minimized *in situ* using the CDOCKER protocol^66^ prior to calculation of binding energies using the distance-dependent dielectric model. All numeration refers to the corresponding mature protein.

## Electronic supplementary material

Supplementary Information
