# Supplementary material for: Novel Molecule Exhibiting Selective Affinity for GABAA Receptor Subtypes
Source: Sci Rep. 2017 Jul 24;7:6230. doi: 10.1038/s41598-017-05966-x (PMC5524711; doi:10.1038/s41598-017-05966-x)

Supplementary Information

Novel Molecule Exhibiting Selective Affinity for $GABA_A$ Receptor Subtypes

Cecilia M. Borghese, Melissa Herman, Lawrence D. Snell, Keri J. Lawrence, Hyun-Young Lee, Donald S. Backos, Lauren A. Vanderlinden, R. Adron Harris, Marisa Roberto, Paula L. Hoffman, Boris Tabakoff

This file contains Supplementary Tables (Tables S1 through S2) and Supplementary Figures (Figures S1 through S6).

Supplementary Table S1. Screening for affinity of DCUK-OEt across receptor/transporter/channel proteins. The listed receptors/transporters/channel proteins demonstrated no significant displacement of specific ligands for these proteins at DCUK-OEt concentrations of 10 μM or less.

Serotonin Rs
5-HT1A
5-HT1B
5-HT2A
5-HT2B
5-HT3
Nicotinic Cholinergic Rs
α2β2
α2β4
α3β2
α3β4
α4β2
α4β4
Adrenergic (β) Rs
β1
β2
Dopamine Rs
D1
D2
Muscarinic Cholinergic Rs
M1
M2

Glycine Rs
$GABA_A$ Rs
ρ1
$GABA_B$ Rs
Histamine Rs
H1
Opiate Rs
μ
δ
Cannabinoid R
CB2
Prostaglandin R
EP2
Vasopressin R
V1A
Metabotropic Glutamate R
mGluR5
Ionotropic Glutamate Rs
NMDA channel binding site
NMDA glycine binding site
Kainate
Serotonin transporter
SERT

Voltage-sensitive Ca^{++} channel (L-type)
Ca_V1.2
Voltage-sensitive Na^+ channels
Brain Na_Vs ([^{3}H]BTX)

Supplementary Table S2. Parameters from GABA concentration-response curves and Zn^{++} effects on EC_{10} GABA responses in different $GABA_A$ receptors. ***n*** represents number of oocytes tested.

Receptor	**EC_{50} GABA (μM)**	**nH**	**n**	**$[Zn^{++}]$ (μM)**	**% change**	**n**
α1β2	1.1 (0.9 to 1.3)	1.19 ± 0.08	5	10	-99 ± 1	5
α1β2γ2	83 (75 to 92)	0.99 ± 0.03	4	10	-9 ± 4*	7
α1β2(N265S)γ2	32 (24 to 42)	0.81 ± 0.06	5	10	10 ± 2*	10
α1β2γ1	21 (16 to 27)	0.87 ± 0.06	6	10	0.4 ± 1.4*	6
α1β3	7.1 (5.7 to 8.8)	0.97 ± 0.06	8	1	-85 ± 3	5
α1β3γ2	15 (13 to 17)	1.33 ± 0.08	4	10	-19 ± 4*	6
α1β3δ	23 (16 to 32)	0.78 ± 0.07	6	1	-35 ± 1*	4
α4β3γ2	12 (9.9 to 14)#	1.01 ± 0.09#	8	1	-43 ± 4*	6
α4β3δ	1.3 (1.0 to 1.7)#	0.84 ± 0.07#	8	1	-31 ± 6*	5
α5β3	4.20 (3.28 to 5.39)	0.93 ± 0.07	6	10	-89 ± 2	8
α5β3γ2	5.64 (4.88 to 6.53)	0.93 ± 0.04	9	10	-29 ± 6*	8
α1β1γ2	30 (20 to 46)	0.76 ± 0.07	4	100	21 ± 3	6

#Borghese et al., 2006

For *EC_{50} GABA*, mean (95% confidence intervals). The rest of the data represent mean ± SEM.

*p≤ 0.0001 versus corresponding αβ; one-way ANOVA followed by Dunnett's multiple comparisons test.

The decreased sensitivity to GABA and/or Zn^{++} inhibition in trimeric $GABA_A$ receptors compared to αβ receptors indicates that the third subunit (either γ or δ) has been expressed and included in the receptors.

Supplementary Figure S1. Representative tracings of EC10 GABA responses in the presence and absence of 0.3 μM DCUK-OEt.

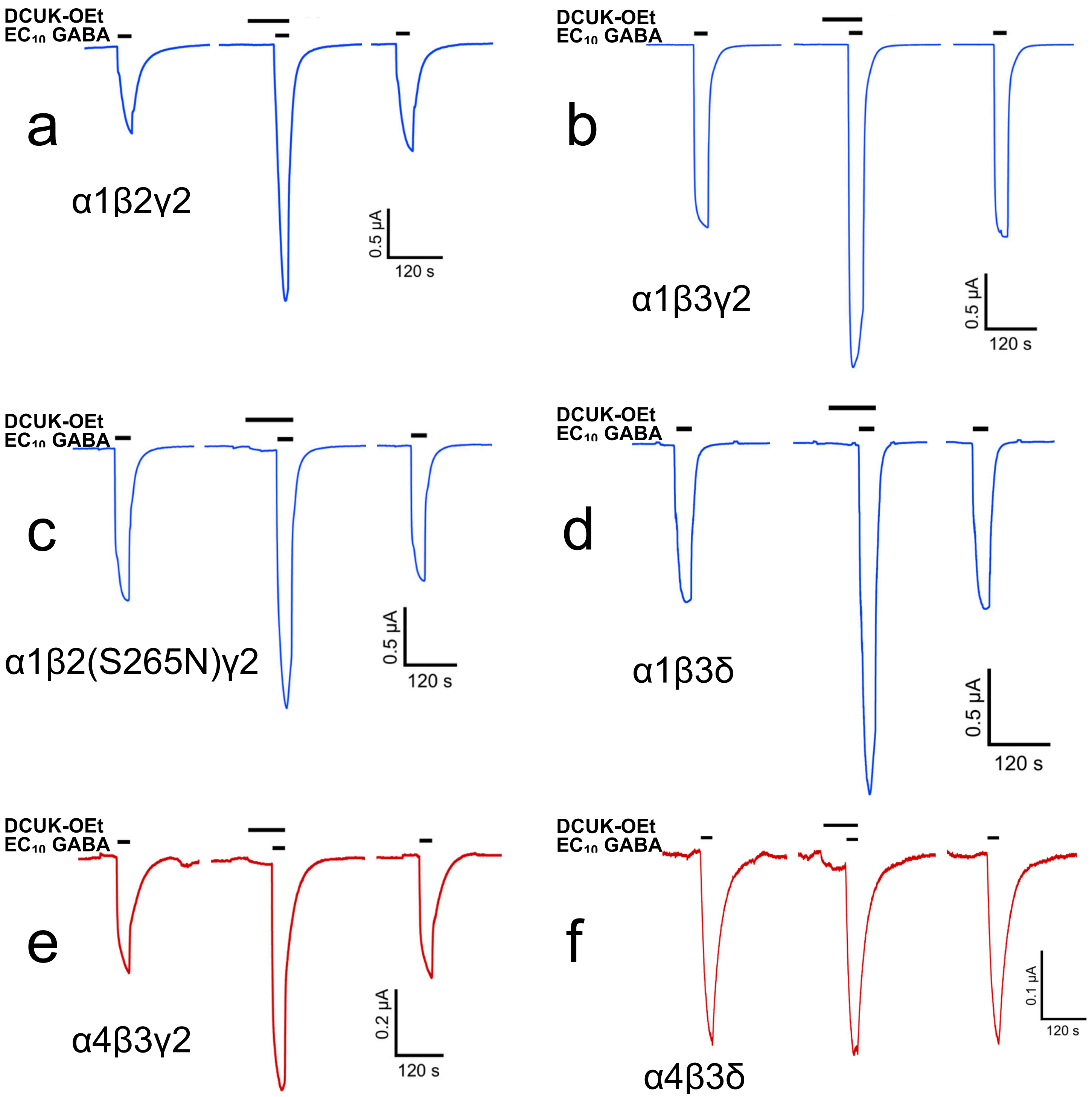

Supplementary Figure S2. DCUK-OEt effect on submaximal (EC_{10}) responses on ρ1 $GABA_A$ receptors and α1 Glycine receptors. Concentration: 0.3 μM DCUK-OEt. Data represent mean ± SEM, n=5. *$p < 0.05$ compared to no DCUK-OEt (t-test).

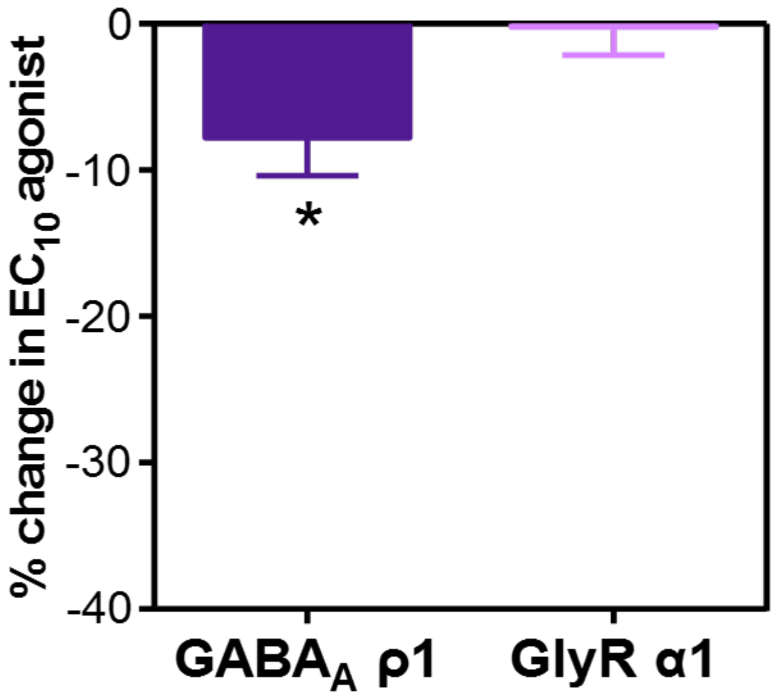

Supplementary Figure S3.17PA effect on DCUK-OEt potentiation of submaximal (EC_{10}) GABA responses in α1β2γ2 $GABA_A$ receptors. **a.** Effects of DCUK-OEt (0.3 μM) and 17PA [17-phenyl-3α, 5α)-androst-16-en-3-ol), 10 μM] alone and co-applied (n=6). **b.** Effects of allopregnanolone (0.3 μM) and 17PA (10 μM) alone and co-applied (n=4). Note difference in X-axis. Data represent mean ± SEM, *p< 0.05, **p< 0.01, repeated measures one-way ANOVA followed by Sidak's multiple comparisons test.

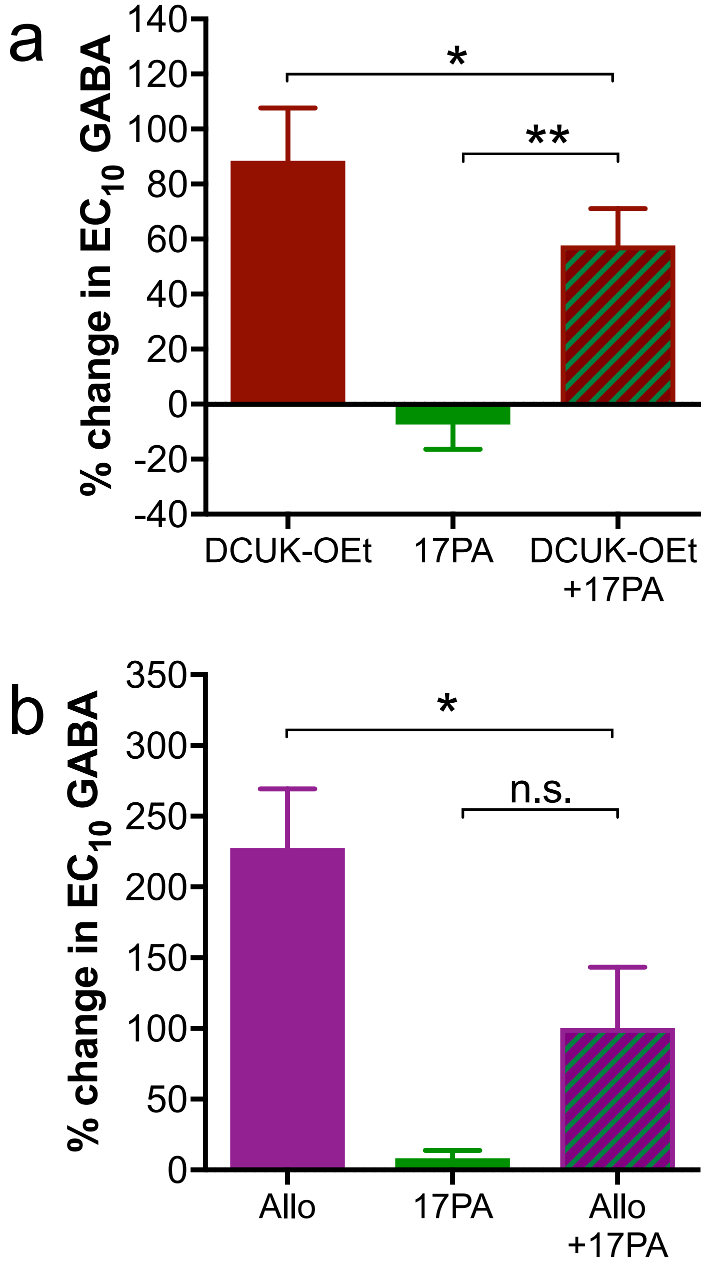

Supplementary Figure S4. Predicted binding of flunitrazepam and etomidate within the extracellular domain of $GABA_A$ receptor subunit interfaces. The α subunit is shaded in green, β in cyan and γ in yellow. The interfaces illustrated were α+β- (alternative site) and α+γ- (benzodiazepine site). Flunitrazepam is represented by gray sticks and etomidate is represented by white sticks. **a.** Flunitrazepam and **b.** etomidate in the alternative site. **c.** Flunitrazepam and **d.** etomidate in the benzodiazepine site. Dashed lines indicate predicted non-bond interactions (green = H-bonds, orange = electrostatic or π-cation/anion, magenta = π-π, purple = π- σ, pink = hydrophobic).

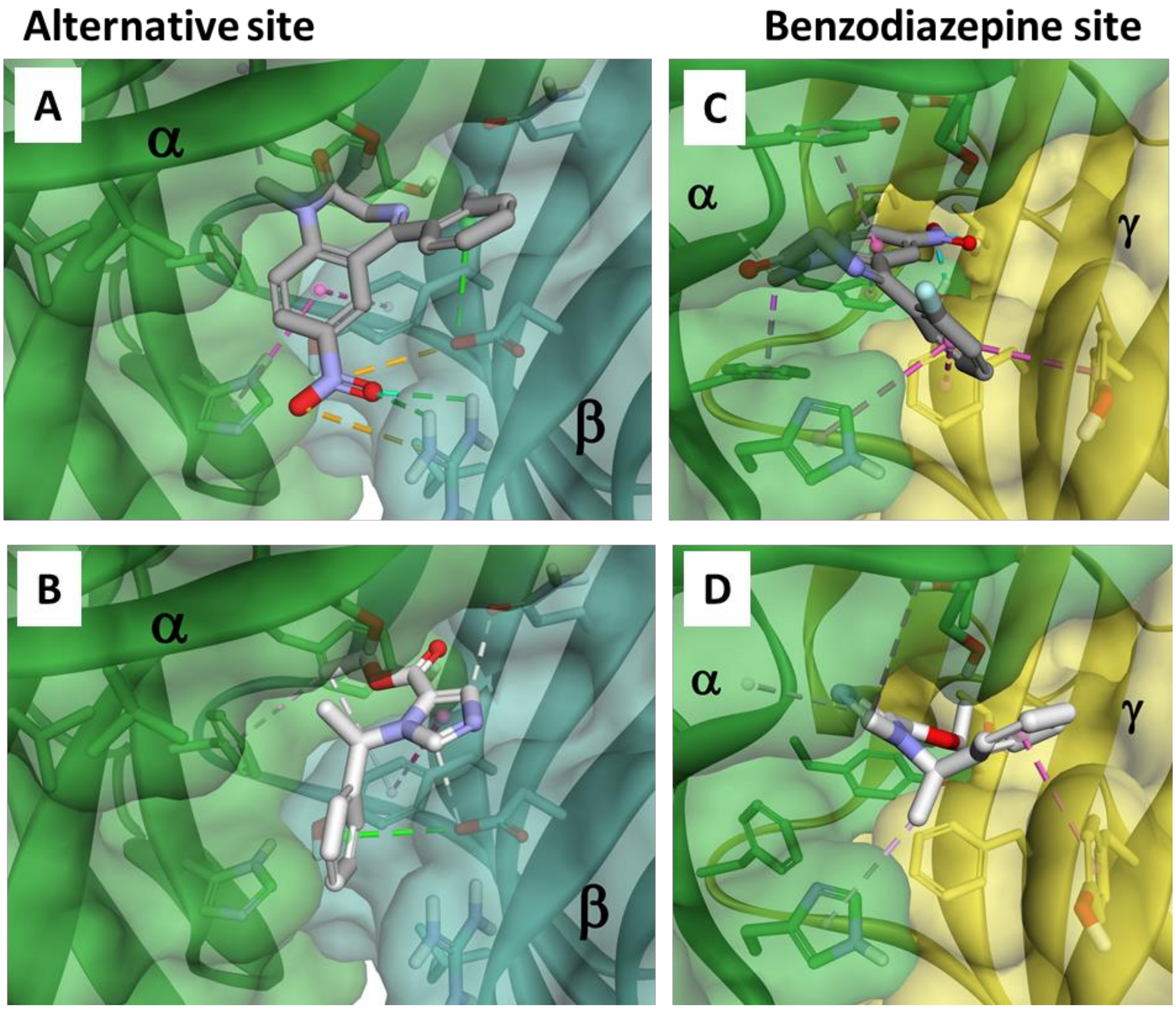

Supplementary Figure S5. Effect of DCUK-OEt and flunitrazepam on submaximal (EC_{10}) GABA responses in α1β2γ2 $GABA_A$ receptors. EC_{10} GABA was determined as described in the Methods section. Before and after each drug+GABA application, EC_{10} GABA was applied and the responses were used as controls for calculating the percentage change. **D**: 1 µM DCUK- OEt pre-applied alone, then co-applied with EC_{10} GABA. **F**: 0.1 µM flunitrazepam co-applied with EC_{10} GABA. **D+F**: 1 µM DCUK-OEt pre-applied alone, then co-applied with EC_{10} GABA and 0.1 µM flunitrazepam. The order of the drug+GABA applications was shuffled for different oocytes. **Sum**: sum of DCUK-OEt and flunitrazepam individual effects. Data represent mean ± SEM, n=7. *p<0.05 compared to the sum of the individual effects (paired t-test).

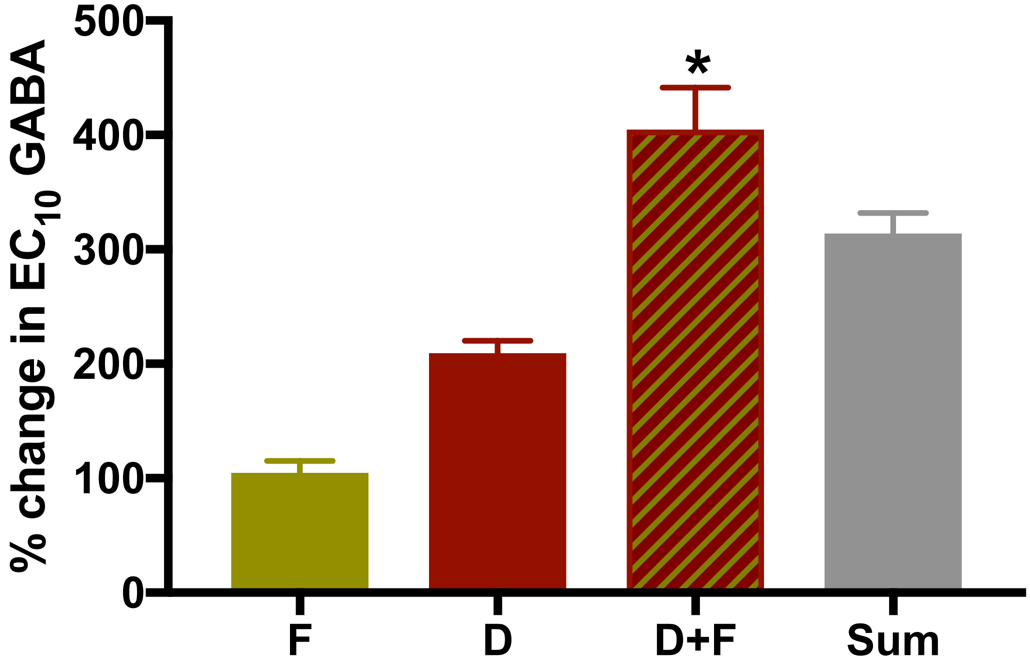

Supplementary Figure S6. GABA concentration-response curves for different $GABA_A$ receptors. **a.** α1β2, α1β2γ1 and α1β1γ2 (n= 4-5). **b.** α1β3, α1β3γ2 and α1β3δ (n= 4-8). **c.** α5β3 and α5β3γ2 (n=6-9). **d.** α1β2γ2 and α1β2(N265S)γ2 (n=4-5). Curves for α4β3γ2 and α4βδ3 already published (Borghese et al., 2006).

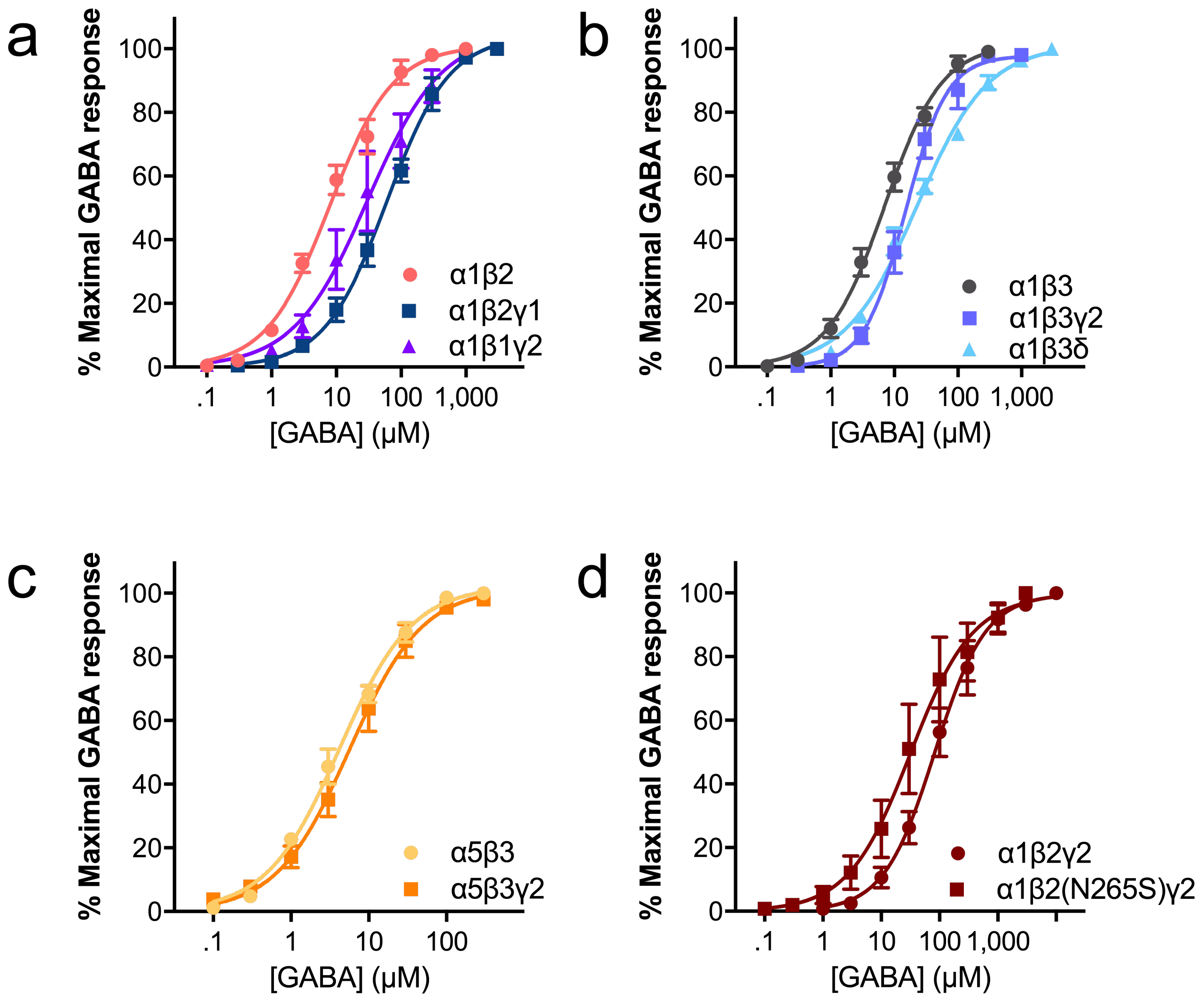

Supplement: Supplementary file 1 — Supplementary Information [file 41598_2017_5966_MOESM1_ESM.pdf]
